# Lack of harmonisation in immunological data: challenges in synthesising data during the COVID-19 pandemic

**DOI:** 10.1016/j.ebiom.2026.106204

**Published:** 2026-03-10

**Authors:** Nicole Shaver, Caroline Colijn, Jane Heffernan, Gideon Darko Asamoah, Thomas Piggott, Curtis Cooper, Salman Bagheri, Angela M. Crawley, Benjamin M. Kagina, Dawn M.E. Bowdish, Marc-André Langlois, Julian Little

**Affiliations:** a Knowledge Synthesis and Application Unit, School of Epidemiology and Public Health, Faculty of Medicine, University of Ottawa, Ottawa, Ontario, Canada; b Faculty of Science, Simon Fraser University, Burnaby, British Columbia, Canada; c Centre for Disease Modelling, Department of Mathematics & Statistics, Faculty of Science, York University, Toronto, Ontario, Canada; d Institute of Clinical Trials and Methodology, University College London, UK; e Department of Health Research Methods, Evidence and Impact, McMaster University, Hamilton, Ontario, Canada; f Peterborough Public Health, Peterborough, Ontario, Canada; g Department of Family Medicine, Queens University, Kingston, Ontario, Canada; h Faculty of Medicine, University of Ottawa, Ottawa, Ontario, Canada; i Ottawa Hospital Research Institute, Ottawa, Ontario, Canada; j Inflammation and Chronic Disease Program, Ottawa Hospital Research Institute, Ottawa, Ontario, Canada; k Canadian Biobank Alliance, Ottawa, Ontario, Canada; l Vaccines for Africa Initiative and NITAGs Support Hub (NISH), School of Public Health, University of Cape Town, South Africa; m Faculty of Health Sciences, McMaster University, Hamilton, Ontario, Canada

**Keywords:** Covid-19, Vaccine effectiveness, Immunogenicity, Assays, Harmonisation, Standardisation

## Abstract

The COVID-19 pandemic drove the rapid development of assays to ascertain immune responses, and laboratories were required to adapt to difficult and quickly changing circumstances. While flexibility and innovation were essential, they also introduced heterogeneity in methods, reagents, and reporting practices between labs. This lack of harmonisation made it difficult to compare findings across studies, slowing evidence synthesis, and limiting the usefulness of data for modelling efforts and policy guidance. Drawing on our team's experience synthesising and modelling vaccine immunogenicity data during the pandemic, we discuss the long-term challenges of standardising human immunology research that were highlighted by the COVID-19 pandemic. We argue that vaccine immunogenicity studies require standardised reporting and quality assessment tools. We propose practical solutions to support comparability of laboratory-based practices, while preserving methodological diversity. By implementing changes before the next public health crisis, future research can avoid waste, strengthen certainty, and maximise policy and practice impact.

## Introduction

Immunological data, including antibody levels and cellular responses, are important for understanding vaccine effectiveness (VE). This is particularly true where immune correlates of protection are known. While clinical outcomes, such as VE against infection or severe disease, are of primary interest to public health professionals and the public, studying immunogenicity may provide additional insights into protective and durable immune responses.^1^ Immunogenicity refers to the ability of a substance to induce an immune response, and may be measured in several ways, including quantifying antibodies or cellular responses directed against target elements (e.g. proteins or peptides), as well as examining the functional capacity to neutralise or eliminate a target, pathogen or pathogen byproduct.

Immunogenicity studies can provide timely, mechanistic insight into vaccine-induced immune protection and are often the primary evidence available for assessing population immunity and immune responses against emerging pathogen variants. Studying immunogenicity was critical during the accelerated process of SARS-CoV-2 vaccine evaluations during the COVID-19 pandemic.^2^ However, many studies focused on the “general” population and were underpowered for underrepresented populations, some of whom could experience different immune response capacities. For example, study participant eligibility criteria for early phase II COVID-19 vaccine trials excluded immunocompromised patients. Meanwhile, prospective cohort studies reported lower antibody responses following vaccination in people with haematologic malignancies or other immunocompromising conditions.^3^ Additional vaccinations (i.e., boosters) in these populations increased antibody titres to levels equivalent to healthy controls and in many regions these individuals were eligible for booster vaccinations based on immunologic data. Immunobridging studies have also been useful to advance vaccine development in other infectious diseases,^4^ including pneumococcal,^5^^,^^6^ seasonal influenza,^7^, ^8^, ^9^ and human papillomavirus (HPV).^10^, ^11^, ^12^ One example is an HPV immunobridging study in which immunological markers were used to infer VE of a single vaccine dose in young girls, a group for which it had been difficult to accrue evidence on HPV infection endpoints.^10^

Despite the rapid accumulation of VE immunogenicity research during the COVID-19 pandemic, there were challenges in consolidating these data to inform mathematical modelling efforts and public health decision-making. In this personal viewpoint, we discuss the considerable heterogeneity, diversity, and lack of harmonisation in immunological data. We focus on vaccine immunogenicity studies (investigations of immune responses following COVID-19 vaccination), rather than clinical effectiveness studies or seroepidemiologic surveys assessing population-level exposure or immunity. These challenges are informed, in part, by our experience conducting a living systematic review and modelling project to examine immunological outcomes in COVID-19 vaccine effectiveness studies.^13^^,^^14^ We highlight challenges that reflect system-level and contextual constraints during a global pandemic, rather than deficiencies in the scientific rigour or intent of individual laboratories or public health actors. We also discuss implications for evidence synthesis and modelling efforts, potential harmonisation approaches and a call to action for coordinated efforts before the next public health emergency. This commentary is intended to guide a diverse audience-including laboratory scientists, funders, journal editors, and health policymakers–toward actionable steps to improve the comparability, reproducibility, and utility of immunological data in the context of future vaccine evaluation and public health decision-making.

## Lack of harmonisation of laboratory measures

Our team conducted living evidence synthesis between January 2020 and April 2023 to examine COVID-19 VE against variants of concern for immunological outcomes, including neutralisation antibody activity, anti-SARS antibody titres, and cell-mediated immune response (methods and results reported elsewhere^14^). Despite the inclusion of over 200 studies that met our eligibility criteria for our living evidence synthesis, our team encountered considerable difficulty in pooling and synthesising data due to multiplicity and lack of comparability of laboratory measures.

A key source of heterogeneity encountered in our review, as in others,^15^ was variability across immunological assays, including choice of target antigen (e.g., spike or nucleocapsid protein, receptor binding domain) and detection technology. The most commonly reported assay types were general neutralising assays, followed by antibody-binding assays, including enzyme-linked immunosorbent assays (ELISA), fluorescence immunoassays (FIA), chemiluminescence immunoassays (CLIA), and multiplex immunoassays. Neutralisation assays may add value when compared to other assay types for assessing functional antibody responses as they evaluate biological activity across the viral replication process.^16^^,^^17^ While results from different neutralisation assays may not be pooled due to methodological and readout differences, comparative laboratory studies can assess relative performance and support cross-assay interpretation.^18^ In our review, we identified 29 distinct neutralisation assays, which we broadly grouped into categories based on the virus system used including assays involving live virus neutralisation (e.g., plaque reduction, focus reduction, microneutralization), pseudovirus neutralisation, and surrogate virus or protein-based neutralisation. While live virus assays are often considered the gold standard, they require biosafety level 3 (BSL-3) containment and are labour intensive,^19^ whereas pseudovirus or surrogate assays offer more feasible alternatives due to lower biosafety requirements and ease of assay execution.

Assays also varied widely in reporting units and metrics, with over 22 different types of neutralising antibody titre unit measures reported across included studies. These included titre-based units that differed based on dilution factors and statistical measures (e.g., geometric mean titres, means, medians). Quantitative readout units (e.g., AU/mL [arbitrary concentration units/mL], IU/mL [international units/mL], and BAU/mL [binding antibody units/mL]) were commonly used but were not always comparable across platforms due to differences in assay calibration. Additional metrics, such as AUC for neutralisation potency and RLU [relative light units], and reporting of log transformations or of relative fold changes, were also used, further complicating data synthesis.

Although increasingly recognised as important for protection against severe disease and long-term immunity for several pathogens,^20^^,^^21^ fewer included studies measured cellular mediated immunity. The relative lack of such studies may be due to the barriers to conducting laboratory cellular immunity assays and the smaller number of T-cell assays that received regulatory authorisation for clinical use during the COVID-19 pandemic.^22^ Such barriers may include additional costs and complexities, including the infrastructure necessary to prepare viable peripheral blood mononuclear cells (PBMCs) of consistent quality, their storage and distribution between study sites and handling procedures for cellular assays.^23^ Among the studies that were included, we found similar issues in standardisation to those for neutralising and antibody-binding assays, with a range of cell-mediated immunity outcomes (e.g., CD4+ T cells vs. CD8+ T cells, B cells), assay types, functional readouts (e.g., IFN-γ+ cells, IFN-γ secretion, antibody-expressing cells) and reported units. Beyond technical barriers, the absence of standardised protocols for cellular immunoassays—such as cytometry gating strategies, cell culture stimulation conditions, and methods for PBMC thawing—contributes to poor comparability across sites. The field also lacks widely accepted qualification/validation criteria and simple pass/fail controls that would permit rapid acceptance of results across laboratories.^24^ Establishing reference protocols, or designating harmonisation hubs for assay validation, could improve the reproducibility of T cell-based and B cell-based immune metrics^23^ and may increase the usefulness of cell-mediated immunity data for population-level decision-making.

This multiplicity of reported measures across studies is unsurprising, as the COVID-19 pandemic required the urgent development of laboratory assays for SARS-CoV-2 immune response assessment. This methodological diversity was both expected and necessary under such emergency conditions, as laboratories rapidly responded with locally available resources before shared standards could be established. In the earliest months of 2020, no validated nucleic acid amplification or serological tests were available for commercial or clinical use, which, alongside infrastructure barriers, led to many laboratories developing in-house assays with whatever local resources were available. The rapidly changing state of knowledge on immunological markers during this period must be considered when interpreting the heterogeneity observed across immunogenicity studies in evidence synthesis. The total number of existing SARS-CoV-2 serological assays is unknown, but estimates are available for commercially available and regulated assays, which proliferated rapidly during the pandemic.^25^^,^^26^ As of March 2025, global assay tracking data indicate that over 2000 regulated COVID-19 assays have been listed from almost 1000 different manufacturers, reflecting the rapid expansion and diversity of diagnostic tests developed during the early pandemic.^27^ Further contributing to inter-laboratory variability were differences in the samples used (e.g., PBMCs vs. whole blood), sample collection methods and sample handling (e.g., plasma vs. serum, haemolysis, freeze–thaw cycles). As discussed in the next section, many of these items were not clearly reported among the included studies in our review. While this proliferation of assays was essential to meeting global testing demands during the pandemic, both the technical differences in the assays (e.g., differences in assay format, antigen targets, analytical sensitivity/specificity) and the heterogeneity in reported metrics (e.g., inconsistent output metrics, unit conventions, positivity thresholds) pose challenges for evidence synthesis.

## Issues in reporting and quality assessment tools

In addition to heterogeneity in laboratory methods, the critical evaluation and integration of immunogenicity data from vaccine effectiveness studies was further complicated by incomplete reporting. To date, there remains a lack of validated risk of bias (RoB) tools aimed at quality assessment in VE immunogenicity studies. Regardless of outcome, VE studies are susceptible to unique issues and biases as distinct from other observational studies,^28^^,^^29^ as VE estimates are particularly sensitive to timing and changes in community transmission, testing access, and public health policies can bias results and confound comparisons over time. Additional complexity was introduced during the COVID-19 pandemic, with issues such as staggered vaccine roll-out, hybrid immunity and emerging variants. For example, COVID-19 VE was often assessed against newer, unmatched variants (i.e., when the antigens in the vaccine did not match those present on circulating SARS-CoV-2 variants), which could have misrepresented cross-protection and limited interpretability.^30^^,^^31^

An additional, often unreported, source of variation in VE immunogenicity studies is the timing of blood sample collection relative to vaccination or infection. Immunological responses vary significantly depending on the number of days post-exposure or post-vaccination, with peak antibody levels and memory B/T cell activation typically occurring in specific intervals.^32^ Beyond timing, pre-analytical factors such as the type of biological sample (e.g., serum vs. plasma), use of anticoagulants, time from collection to processing, storage temperature and duration, and the number of freeze–thaw cycles can also affect assay performance and measurement reliability. These factors influence antibody stability, protein integrity, and cell viability, contributing further to inter-study variability and an inability to interpret the findings if not clearly reported. Including standardised time points, sample handling protocols, and stratified reporting would support the reproducibility and interpretability of immune response data.

The WHO released interim guidance for post-introduction VE evaluations in 2021 to address some of these issues.^33^ However, to our knowledge, no guidance is available for biases particular to immunological data. Therefore, to supplement our quality appraisals using existing RoB tools, our systematic review applied the ROSES-I (Reporting of Seroepidemiologic Studies for Influenza) statement,^34^ an extension of STROBE developed by a consensus process for influenza seroepidemiologic studies, given the absence of a validated reporting guideline specific to COVID-19 at the time of protocol development. Later, in June 2021, an updated version of the ROSES-I statement was released (ROSES-S: Reporting of Seroepidemiologic studies–SARS-CoV-2) for application to seroepidemiologic studies conducted during the COVID-19 pandemic.^35^ However, the items in both statements are very similar, as the ROSES-S expands on the domains in the ROSES-I statement.

Using the ROSES-I extension, we found that only 64 of 212 studies (30%) adequately reported on items 11.1–11.3,^34^ which pertain to the handling of quantitative variables, including assay limits of detection (LoD) and treatment of values below the LoD, definitions of seropositivity and seroconversion, and correlation with protection. Notably, 94% of included studies failed to report on ROSES-I items 12.a.1–12.a.14,^34^ including qualifying information about laboratory methods, including the sample type and specimen storage conditions, assay type and endpoint determination, cross-reactivity assessment, and reference to a standardised protocol. Many studies did not document which viral strains, spike protein constructs, or assay protocols were used for variant-specific neutralisation, further complicating cross-study comparisons. These findings align with a recent report that found suboptimal reporting in SARS-CoV-2 seroepidemiologic studies using the ROSES-S guideline, with the laboratory methods domain having the lowest median adherence.^36^ It should be noted that, while these reporting gaps limit interpretability and the ability to pool results, they do not necessarily imply that the underlying assays or findings were scientifically unreliable.

Finally, many studies failed to report on the qualification (i.e., establishing that an assay is suitable for its intended purpose) and validation status (i.e., performance characteristics) of assays. The accurate and sensitive measurement of antibodies is critical to assess immune response following vaccination, as well as infection prevalence during outbreaks and pandemics. Large-scale SARS-CoV-2 serosurveillance initiatives have demonstrated the importance of using validated assays^37^, ^38^, ^39^ and the need for validated risk of bias tools for trustworthy public health decision-making.^40^ Early in the pandemic, validation and reporting practices were still evolving. In this context, a systematic review of over 1800 serosurveys found that only approximately 28% of commercial assays met WHO criteria for emergency use based on manufacturer-reported data, with lower concordance observed in third-party and independent evaluations.^39^ These findings highlight challenges in assay evaluation and transparency during a rapidly evolving public health emergency and the importance of using and reporting appropriately qualified and validated assays.

## Need for harmonisation in evidence synthesis and modelling

Evidence synthesis and modelling efforts require harmonised immunological inputs. For example, consider three studies evaluating SARS-CoV-2 VE immunogenicity following a second BNT162b2 vaccine dose, one using a commercial receptor binding domain IgG assay reporting BAU/mL, another using an in-house Spike IgG ELISA reporting arbitrary units, and a third reporting neutralisation titres using a live-virus assay. Even if all three suggest a “protective” immune response, the absolute titres are not directly comparable due to differences in assay standardisation, detection limits, and calibration. Moreover, total IgG measurements do not distinguish between IgG subisotypes (e.g., IgG1 vs. IgG4), which can differ significantly in their functional capacity to neutralise the virus.^41^

Such variability has direct implications for interpreting immune correlates of protection. For example, establishing a protective antibody threshold requires consistency in how immune responses are measured and reported to distinguish between biological variation and methodological noise. This heterogeneity also affects durability estimates (i.e., antibody waning kinetics) as changes in titre levels over time may not be reliably compared across studies using different assays. Overall, the inability to pool data across immunogenicity studies reduces statistical power in evidence syntheses and results in less robust conclusions.^42^^,^^43^

These issues also impact modelling efforts. Mathematical models are frequently called upon for scenario projections and explorations of the impacts of potential vaccine policies. Models require assumptions about the effectiveness of vaccination, and model results will differ markedly under different assumptions about the strength and duration of vaccine-induced immunity. Furthermore, the protection conferred against infection (and hence also infectiousness), symptomatic disease, severe disease, and/or Post-Covid Conditions (PCC, aka “long-Covid”) matters for model outcomes and hence for model-aided advice given to decision-makers. While mixed-effects models can adjust for either assay or unit variability, the simultaneous lack of standardisation in both limits the range of scenarios that can be explored. Machine learning models may help to bridge some of these gaps in the future; network approaches have shown promise in helping to understand the complex interplay between age, sex, antibody responses and vaccination throughout the COVID-19 pandemic.^44^

The aforementioned issues demonstrate the longstanding and critical need for harmonisation of laboratory assays and reporting measures/parameters to enable (1) the comparison of vaccine-related data and other immunological data between studies, and (2) the production of powerful data-informed immune system mathematical modelling studies with high predictive power and confidence in results.

## International standardisation and harmonisation efforts during the pandemic

The standardisation of immunological assays has long been recognised as critical for generating comparable and policy-relevant data across other infectious diseases. For example, the National Institute of Biological Standards and Control (NIBSC) and WHO have developed global standardisation efforts for seasonal influenza,^45^ HPV,^46^ and Human Immunodeficiency Virus (HIV),^47^ to provide calibrated reference materials, validated assay protocols, and guidance on correlates of protection (Table 1). The urgent need for international standards for materials to enable calibration between laboratories was recognised early on during the COVID-19 pandemic. The WHO released the first International Standard for anti-SARS-CoV-2 immunoglobulin (NIBSC 20/136) in December 2020,^50^ providing a reference measurement of neutralising activity in international units (IU) and proposing calibration of direct binding antibody assays using binding antibody units (BAU). International Standards also serve as primary reference materials to calibrate secondary or national standards. For example, in the United States, the Frederick National Laboratory for Cancer Research, in collaboration with the National Cancer Institute's Serological Sciences Network (SeroNet), developed the U.S. SARS-CoV-2 Serology Standard as a secondary reference material calibrated to the WHO International Standard. This standard was subsequently implemented across SeroNet laboratories to harmonise assay performance and reporting units.^51^^,^^52^ SeroNet, in collaboration with the WHO Solidarity II initiative, helped to demonstrate the feasibility and utility of harmonising serological data to a global standard.^51^

**Table 1:** Examples of international standardisation and harmonisation initiatives for immunoassays.

Pathogen	Coordinating body (ies)	Initiative description	Available guidance and resources
Influenza	National Institute for Biological Standards and Control (NIBSC)	Reference reagents for seasonal and (pre)pandemic influenza vaccine standardisation, used globally by manufacturers and control laboratories for vaccine release and quality control	NIBSC Influenza Resource and reagent updates (link)
Human papillomavirus (HPV)	HPV Serology Laboratory (HPVSL) (Frederick National Laboratory for Cancer Research, FNLCR), World Health Organization (WHO), National Institute for Biological Standards and Control (NIBSC), HPV LabNet	HPSL leads an HPV Serology Standardisation initiative for standardisation and harmonisation of HPV serology assays^48^ A collaborative effort coordinated with NIBSC and WHO to harmonise HPV serological assays led international serology standards for antibodies to seven HPV types (HPV6, 11, 31, 33, 45, 52, 58)^46^	• HPVSL HPV Serology Standard Operating Procedures (link) • WHO International Standards for Antibodies to Human Papillomavirus Types 6,11, 31, 33, 45, 52 and 58 (link) • HPV Laboratory e-Manual (link)
Human immunodeficiency virus (HIV)	World Health Organization (WHO), National Institute for Biological Standards and Control (NIBSC)	International standards and reference panels for HIV diagnostic assay calibration, including HIV-1 RNA viral load (e.g., NIBSC 16/194), p24 antigen (WHO IS 22/230)	• 4th WHO International Standard for HIV-1 RNA for Nucleic Acid Amplification Techniques (NIBSC 16/194) (link) • 1st WHO International Standard for HIV-1 p24 Antigen (NIBSC 22/230) (link)
SARS-CoV-2	World Health Organization (WHO), National Institute for Biological Standards and Control (NIBSC)	The WHO established WHO International Standards (NIBSC 20/136; 22/270) for antibody and neutralisation assays and leads Solidarity II, a global collaboration to promote the implementation of SARS-CoV-2 serological surveys	• 1st WHO International Standard for anti-SARS-CoV-2 immunoglobulin (human) (NIBSC 20/136) (link) • 1st International Reference Panel for antibodies to SARS-CoV-2 variants of concern (NIBSC 22/270) (link) • WHO “Solidarity II” global serologic study for assay standardisation (link)
SARS-CoV-2	Frederick National Laboratory for Cancer Research (FNLCR) with SeroNet	The U.S. SARS-CoV-2 Serology Standard (secondary reference) calibrated to WHO units was distributed via SeroNet laboratories to harmonise assay performance and reporting; the former COVID-19 Serology Laboratory developed SARS-CoV-2 serology standards and protocols to promote standardisation across SeroNet studies	• SARS-CoV-2 serology operating procedures (link)
SARS-CoV-2	COVID-19 Immunity Task Force (CITF)	The CITF Harmonisation Initiative aimed to harmonise immunological datasets across 40+ CITF-funded Canadian studies, including standardised variables for serology, cellular immunity, vaccination status, and infection history^49^	CITF Harmonisation Initiative portal and metadata documentation (link)

Despite the success of these coordinated initiatives to generate high-quality immunological data standards, several challenges may have slowed their widespread adoption. Concerns were raised regarding the applicability of this standard to emerging variants of concern with greater antigenic drift, such as Delta and Omicron,^51^ resulting in the WHO developing a new reference panel (NIBSC 22/270) that included antibodies targeting newer variants.^53^ Still, inconsistencies remained in how studies measured variant-specific neutralisation, often using different spike protein constructs or viral isolates, which further complicated cross-study comparisons of vaccine-induced immunogenicity. International harmonisation efforts largely focused on IgG, and it became difficult to universally standardise when IgA was found to be a potential correlate of protection. This is a concern in outbreak scenarios, as correlates of protection are unknown at the outset and evolve with each variant.

As with much of the research conducted during the COVID-19 pandemic, factors influencing the lag in WHO International Standard implementation were likely multifaceted and reflected logistical, financial, and temporal constraints faced by laboratories operating under intense pressure. When the WHO released the International Standard in late 2020 and updated standards for anti-SARS-CoV-2 immunoglobulin and variants of concern in 2022, many laboratories had already developed and validated their own assays, protocols, and internal standards. Retrospectively recalibrating assays to align with WHO standards would have required additional validation steps and might have delayed research at a time when rapid results were perceived to be critical for informing public health responses. Re-calibration to WHO standards potentially could have been costly and complex, and many laboratories may have lacked the funding, reagents, or equipment necessary. Additionally, shipping delays and supply chain constraints due to the depletion of the first WHO International Standard affected the availability of WHO reference materials.^51^ To improve future uptake of international standards like those from the WHO, support systems—such as funding mechanisms, reagent-sharing platforms, and training for recalibration procedures—should be built into harmonisation plans. These could help labs overcome logistical and resource barriers that limited adoption during the COVID-19 pandemic.^51^

These barriers likely contributed to the limited adoption of WHO guidance on serological standardisation during the COVID-19 pandemic, resulting in inconsistencies in how immunogenicity data were measured and reported. Had there been more widespread harmonisation (e.g., adoption of common reference standards, assay calibration protocols, reporting units) in even a fraction of the studies released during this time period, immunogenicity data could have been pooled across diverse populations, vaccine platforms, and timepoints to generate more robust estimates of the magnitude and durability of vaccine-induced immune responses, including against emerging variants. These insights would have supported timelier and evidence-based guidance to policymakers in 2021 and 2022 as they weighed decisions on booster campaigns, dose intervals, and variant-specific strategies.

## Call to action

It is important to learn from public health crises to avoid the same issues recurring in subsequent outbreaks.^54^, ^55^, ^56^ Our and other investigators’ experiences conducting research during the COVID-19 pandemic highlighted gaps in assay standardisation, data harmonisation, and transparency of reporting. To address these gaps, a collective and coordinated effort is needed across biobanks, research laboratories, funders, journals, and research institutions. We have summarised some of these challenges, ongoing initiatives and suggestions for actionable solutions in Table 2. Our recommendations are intended to strengthen system-level coordination, infrastructure, and preparedness for future public health emergencies, not to retrospectively evaluate the actions of individual laboratories, regulators, or research teams.

**Table 2:** Summary of areas for standardisation and harmonisation in immunological data.

Phase	Areas for standardisation or harmonisation	Developments during COVID-19	Challenges identified	Proposed solutions/Ongoing efforts
Sample collection, processing and storage	• Consideration of timing of blood draw (e.g., post-infection or vaccination, relation to symptom onset) • Specimen type (e.g., serum vs. plasma) • Use of anticoagulants • Processing time after collection • Freeze–thaw cycles • Storage temperature	• ISBER–BBMRI Joint COVID-19 Biobanking Initiative (2020–2021) focused on harmonising biospecimen availability and metadata^57^	• Variable collection timing • Difficulty of transport conditions • Limited adherence to biobanking SOPs • Underreporting of storage conditions	• Develop unified collection protocols • Develop assay-specific pre-analytical quality criteria • Promote ISBER standard operating procedures for specimen handling^58^ • Expand training workshops and accreditation for biospecimen management
Assay testing and validation	• Assay qualification and validation procedures • Use of standardised reference materials **Antibody-Binding Assays** • Antigen selection • Cut-off determination **Neutralisation Assays** • Reference virus strain or spike variant • Readout metric (e.g., PRNT50, IC50, AUC) **Cell-Mediated Immunity** • PBMC isolation • Stimulation protocols • Gating strategies • Cytokine readouts	• Establishment of international and secondary standards for SARS-CoV-2 for antibody and neutralisation assays (e.g., NIBSC 20/136,^51^ U.S. SARS-CoV-2 Serology Standard^52^)	• Inconsistent reporting of qualification/validation status • Lack of consensus on minimum performance criteria • Limited availability of validated reference panels **Antibody-Binding Assays** • Heterogeneous assay formats and targets • Inconsistent unit reporting • Differences in cut-offs **Neutralisation Assays** • Need for BSL-3 facilities for live-virus assays • Limited standardisation of pseudovirus constructs **Cell-Mediated Immunity** • Lack of universally accepted validation criteria • Logistical barriers for viable cell handling	• Promote use of qualified and validated assays in immunogenicity and VE studies • Consider inter-laboratory proficiency testing and external quality assessment (EQA) • Develop centralised repositories of validated assays and reference materials • Establish dedicated funding and reagent-sharing programs to facilitate assay recalibration to international standards • Provide training and technical support to laboratories adopting new reference materials **Antibody-Binding Assays** • Broader adoption of WHO-calibrated standards • Open-access calibration protocols **Neutralisation Assays** • Harmonise reporting of neutralisation potency using standard units (IU/mL or equivalent) **Cell-Mediated Immunity** • Establish reference protocols for cell-based immune metrics • Create harmonisation hubs for assay validation • Develop simple pass/fail controls and reference reagents
Assay readout	• Units • Thresholds • Calibration to international or national standards	• WHO International Standard (IU, BAU/mL)	• Inconsistent calibration to standards • Missing LoD, LoQ, and seroconversion definitions	• Promote reporting of calibration units and performance parameters • Develop shared vocabularies and controlled terminology
Data sharing and integration	• Metadata and definitions across cohorts • Development of interoperable data infrastructures for immunological and VE studies	• ROSES-S Statement from the World Health Organization on the reporting of seroepidemiologic studies for SARS-CoV-2^35^ • CITF Harmonisation Initiative established core data elements and data dictionary for immunology and VE studies^49^ • CanPath COVID-19 Neutralisation Serology Harmonisation developed a protocol to generate harmonised datasets across the population-based cohorts^59^	• Heterogeneity of variable definitions and metadata	• Develop harmonised core data element sets • Use common data dictionaries • Require structured data sharing • Incentivise data deposition in harmonised repositories • Encourage journals and repositories to enforce reporting guidelines
Knowledge synthesis and modelling	• Evidence synthesis of immunogenicity data • Risk of bias assessment of immunological data and VE data • Modelling using immunological correlates	• SeroTracker global dashboard and data harmonisation platform consolidating and assessing quality of SARS-CoV-2 seroprevalence data^40^	• Heterogeneity in assay format, antigen targets, and analytical sensitivity • Poor adherence of individual studies to reporting templates • Lack of validated quality assessment tools for immunogenicity research • Unknown correlates of protection early in the pandemic	• Develop machine-learning and statistical harmonisation models to bridge assay gaps • Apply living evidence synthesis frameworks for evolving knowledge • Create standardised bias-assessment tools for immunological VE studies • Publish protocols for uncertainty and sensitivity modelling of immunogenicity data
Systems-level infrastructure and data integration	• Global and national coordination networks • Coordination and harmonisation of COVID-19 research in laboratories through biobanking infrastructures	• ISBER–BBMRI Joint COVID-19 Biobanking Initiative (2020–2021) focused on harmonising biospecimen availability and metadata coordinated international biobanking initiatives to strengthen research capacity during the COVID-19 pandemic^57^ • Canadian Biobank Alliance (CBA) national biobanking network supported harmonised sample processing and data sharing through the Universal Data and Biological Materials Transfer Agreement (UDBMTA)^60^^,^^61^	• Fragmented infrastructure and siloed data systems • Lack of regulatory coordination early in outbreaks • Limited integration between biobanks and immunological assay development • Lack of interoperable metadata standards for biospecimen annotation	• Support cross-platform interoperability through shared metadata frameworks • Leverage biobanking infrastructures to facilitate specimen exchange and harmonised biorepository management during outbreaks, including data sharing agreements

While offering suggestions for standardisation and harmonisation, we must first emphasise that heterogeneity is an inevitable feature of emergency science. The goal of harmonisation should not be the uniformity of methods but the comparability of outputs. Scientific efforts should allow for flexibility, particularly during pandemic scenarios, where some methodological diversity and evolution may be both unavoidable and beneficial. Indeed, overly rigid guidelines risk stifling the creativity that proved essential early in the pandemic, when the immunological correlates of protection were still unclear. Diverse, independently developed assays enabled rapid progress and helped the field converge more quickly on meaningful immune markers. As seen with shifts in anti-N antibody dynamics during the Omicron era, adaptability was key and standardised tools had to be recalibrated as new evidence emerged. A flexible approach to harmonisation can preserve innovation, nimbleness and speed, while still enabling comparability across studies.

In principle, global regulatory agencies would provide assay-related guidance on laboratory standardisation as early as possible in outbreak scenarios to support the transition from research settings to clinical practice. While regulatory agencies acted swiftly to authorise the use of diagnostic tests during the COVID-19 pandemic, the initial absence of standardised assay guidance and global coordination led to inconsistencies in testing practices. Academic networks (e.g., in Canada: Coronavirus Variants Rapid Response Network, CoVaRR-Net; and the COVID-19 Immunity Task Force, CITF) may be able to operationalise rapidly, and can serve as central hubs for assay calibration and methodological alignment. These efforts could be supported by leveraging established data harmonisation platforms for population-based research such as DataSHaPER,^62^ Maelstrom Research,^63^ and the BioSHaRE project^64^ as well as following standard operating procedures for specimen handling promoted by the International Society for Biological and Environmental Repositories (ISBER).^58^

Harmonisation efforts and rapid knowledge exchange during public health crises may be supported by global and regional biobanking networks, as observed during the COVID-19 pandemic. One such example was the Biobanking and BioMolecular resources Research Infrastructure—European Research Infrastructure Consortium (BBMRI-ERIC) partnering with ISBER to coordinate international biobanking initiatives to strengthen research capacity during the COVID-19 pandemic.^57^^,^^65^ In Canada, such harmonisation efforts were supported by a national academic biobanking network system, the Canadian Biobank Alliance (CBA). Coordination between laboratories enabled harmonisation of antibody measurements across blood and dried blood spot samples using WHO standards.^60^ Following the pandemic, the CBA has initiated efforts to promote harmonisation of laboratory practices across its national network. These include developing standardised protocols for specimen processing, storage, and documentation to improve consistency and facilitate reproducibility. Critical to knowledge exchange among laboratories, the CBA pioneered a universal data and biological materials transfer agreement (UDBMTA),^61^ which helped to catalyse broader data-sharing initiatives in Canada.^66^ As these frameworks evolve, they offer a model for integrating harmonised biospecimen management into immunological assay pipelines, especially for large-scale studies.

While the universal standardisation of laboratory-based practices is not feasible or desirable, regulatory bodies, clinical trial sponsors and funding agencies should continue to strive to promote crosstalk between laboratories, modellers, and other stakeholders. Promoting the sharing planned research protocols, aligning assay calibration approaches, and using common reference standards (e.g., IU, BAU) may also help to improve comparability while preserving methodological diversity and allowing labs to use their own platforms. Recognising the need for standardising human immunology research, consortia, such as the Canadian Autoimmunity Standardisation Core (CAN-ASC) have championed efforts into the harmonisation of laboratory assays. These efforts include hosting training workshops and standardising protocols for sample collection, processing, freezing, and thawing practices.^67^

One proven approach from clinical laboratory practice that could inform research harmonisation is external quality assessment (EQA). In clinical labs, reference laboratories routinely distribute blinded samples to participating sites to assess inter-laboratory consistency. Adapting such proficiency testing schemes in immunology research settings—particularly those using ELISA, cytometry, or neutralisation assays—could provide a practical mechanism to identify variability in assay performance and improve data harmonisation.^68^

Laboratories should be also supported with practical and highly accessible resources to improve reporting of methods. Endorsement of reporting guidelines such as ROSES-I and ROSES-S could be sought from funding agencies, with adherence to a minimum dataset, made a condition of funding. Journals could also endorse these guidelines and participate in auditing their use to strengthen transparency across research and clinical domains. Complementary efforts, such as promoting common data dictionaries and harmonised metadata standards, would enhance cross-study usability while allowing researchers the flexibility to pursue novel approaches.

Finally, researchers, including systematic reviewers and modellers, should invest resources in developing guidance to support (1) the evaluation and synthesis of heterogeneous immunological data, and (2) the design of new statistical and mathematical modelling frameworks capable to generating meaningful predictions from non-optimal or incomplete data. Tools for statistical harmonisation such as cross-platform calibration models and meta-analysis weighting schemes should be developed and shared. Additional guidance should be developed to address challenges in assessing bias in serological and neutralisation-based VE studies to standardise evidence synthesis efforts. This may include developing new quality appraisal tools or applying existing reporting guidelines to inform appraisal. The rapid technical development of assays and state of immunological knowledge during public health crises (e.g., correlates of protection) also necessitate adaptable frameworks for evidence synthesis. Living systematic reviews are one evidence synthesis approach well suited to such rapidly evolving evidence bases,^69^ enabling refinement of eligibility criteria and quality assessment as assay validation, harmonisation strategies and reporting standards emerge. Finally, protocols for conducting sensitivity and uncertainty mathematical modelling studies should be developed to enhance transparency and reliability of model-based inferences.

## Conclusion

The COVID-19 pandemic demonstrated both the power and the limitations of rapid immunological research conducted under emergency conditions. Researchers across the globe rose to meet a substantial challenge. Those studying immunogenicity demonstrated speed and ingenuity in developing novel assays, generating data in real time, and continuously adapting their methods in response to an evolving virus and shifting public health needs. While assay innovation and methodological diversity were essential for early discovery, our experiences highlight the need for greater coordination, transparency, and harmonisation to ensure that immunogenicity data can be efficiently synthesised and translated into policy-relevant insights. Looking ahead, investment in flexible international standards, assay calibration infrastructure, fit-for-purpose quality assessment tools, and closer integration between laboratory scientists, evidence synthesis teams, and modellers will be critical to strengthening pandemic preparedness. Standardising human immunology research assays has been a long-term issue and encouraging harmonisation requires a proactive and coordinated response from laboratories, funders, regulatory agencies, and academic networks. By embedding these system-level supports, frameworks can support both flexibility in methodological approaches and comparability in immunogenicity data. Let us not now lose the hard learnt lessons of immunological research during the COVID-19 pandemic and prepare for the next public health crisis.

### Living evidence synthesis methods summary

Several iterations of a living systematic review were conducted between January 2020 and April 2023 to examine COVID-19 VE against variants of concern for immunological outcomes, including neutralisation antibody activity, anti-SARS antibody titres, and cell-mediated immune response. We searched electronic databases (MEDLINE; Embase; Cochrane, L∗OVE), pre-print servers, and grey literature. Screening, data extraction and quality assessments (Ottawa Newcastle Scale for observational studies and the Cochrane ROB-2 tool for RCTs) were performed in duplicate. Results were synthesised descriptively due to substantial heterogeneity. A complete summary of our methods, including full search strategy and eligibility criteria, can be found in our protocol.^13^

## Contributors

AMC, BK, CColijn, CCooper, DB, GDA, JH, M-AL, SB, TP: Review protocol development and methodology, formal analysis, validation, interpretation of findings, review and editing.

JLittle: Conceptualisation, funding acquisition, review protocol development and methodology, supervision, interpretation of findings, review and editing.

NS: Conceptualisation, project administration, review protocol development and methodology, data curation, formal analysis, writing of the original draft, and writing—review and revisions.

NS, JLittle, and GDA had full access to all the underlying data, verified the data used in the analyses, and take responsibility for the integrity of the data and the accuracy of the data analysis.

All authors have read and approved the final version of the manuscript and agree to its submission for publication.

## Declaration of interests

JH has received research funding from the Natural Sciences and Engineering Research Council of Canada (NSERC) Discovery Grant program and the New Frontiers in Research Fund—Exploration (NFRF-E). TP has received grant funding from the Public Health Agency of Canada for research on Post COVID-19 Condition and from the World Health Organization for work related to Essential Medicines Lists, both paid to their institution. BK has received independent educational grants from pharmaceutical companies to support the Annual African Vaccinology Course and has received honoraria for presentations on pneumococcal conjugate vaccines, as well as support to attend immunisation symposia related to pneumococcal and rotavirus vaccines. DB has received consulting fees from Pfizer Global, GSK Canada, and AstraZeneca Global for work related to COVID-19, pneumococcal, and other vaccines; has participated in an advisory board for Pfizer on COVID-19 vaccines; and provided expert witness testimony on vaccine-related matters in June 2024. DB also serves as an unpaid member of the Board of Directors of the Lung Health Foundation. JL has received research funding from a Canadian Institutes of Health Research (CIHR) operating grant and the Coronavirus Variants Rapid Response Network, both paid to their institution. All other authors declare no competing interests.
